# Participant and Provider Perspectives on a Novel Virtual Home Safety Program for Fall Prevention in Parkinson’s Disease

**DOI:** 10.3390/jcm14145031

**Published:** 2025-07-16

**Authors:** Mitra Afshari, Surabhi P. Dharmadhikari, Vijay G. Palakuzhy, Andrea V. Hernandez, Alison W. Hauptschein, Christopher G. Goetz

**Affiliations:** 1 Department of Neurology and Rehabilitation, University of Illinois at Chicago, Chicago, IL 60612, USA; sdharm3@uic.edu (S.P.D.); vpalakz@uic.edu (V.G.P.); 2 School of Medicine and Public Health, University of Wisconsin—Madison, Madison, WI 53705, USA; avhernandez5@wisc.edu; 3 Department of Physical Therapy, Rush University Medical Center, Chicago, IL 60612, USA; alison_hauptschein@rush.edu; 4 Department of Neurological Sciences, Rush University Medical Center, Chicago, IL 60612, USA; christopher_goetz@rush.edu

**Keywords:** Parkinson’s Disease, telehealth, falls, patient perspectives

## Abstract

**Background/Objectives**: Telehealth enhances access to specialty care, but stakeholder perspectives are often overlooked. The objective was to evaluate participant and provider satisfaction with a novel telehealth-enabled home safety program. **Methods**: This is a sub-investigation of a non-randomized pilot study of a novel telehealth-enabled home safety program that enrolled 23 persons with Parkinson’s Disease (PwPs) at risk for falls and their respective care partners (CPs). Dyads participated in four to six televisits over three months, where they performed “virtual home tours” using a mobile platform (tablet mounted on a rolling stand) with a physical therapist to identify and mitigate environmental fall hazards. Satisfaction was assessed using PI-developed surveys and open feedback. Mobile platform usability was assessed with the System Usability Scale (SUS). **Results**: A total of 95.65% of dyads were *very* to *extremely satisfied* with the entire program overall, and the therapist indicated the same for 73.91% of the dyads. Additionally, 95.65% of dyads reported gaining new awareness of home fall hazards. Difficulties maneuvering the mobile platform, using a tablet, and connectivity issues were common challenges noted. The mean score on SUS for the mobile platform was 65, indicating poor perceived usability, and most dyads indicated they would have preferred using a smartphone for the program. Other priorities, including competing health and personal obligations, along with resistance to change, were the primary barriers to implementing program recommendations. **Conclusions**: Our novel telehealth-enabled home safety program was well-received by patients and the study therapist. Using a smartphone and troubleshooting connectivity issues might help further improve the usability and accessibility of this program.

## 1. Introduction

Home-based telerehabilitation programs have emerged as a novel approach to tackling gait and balance dysfunction in Parkinson’s Disease (PD), enabling remote assessment and rehabilitative intervention delivery directly to patients in their homes by more specialized therapists [1,2,3,4]. In addition to improving patient access to PD-specialized therapists, “virtual” home-based programs provide the unique advantage of real-time assessment of the patient in their home, the home environment itself, and optimally tailored recommendations to overcome gait and balance issues and prevent falls [1,2,3,4].

While numerous studies have explored the feasibility and efficacy of novel telerehabilitation programs for PD patients, studies that primarily highlight participant and provider perspectives on these programs are sparse. While technology-enabled care has many benefits, it is essential to ensure that it aligns with participant and provider experiences and expectations to promote meaningful engagement and adherence. Assessing how participants and providers interact with technology and telehealth platforms is critical for designing novel patient-centered health services. Both quantitative usability metrics and qualitative feedback are essential to ensure these interventions meet the needs and expectations of both stakeholders.

In this sub-investigation of a pilot non-randomized study, we place the participants’ and providers’ voices at the center and assess their satisfaction and technological preferences with respect to a *novel virtual home safety program* that targets environmental fall risk factors in the home for persons with Parkinson’s Disease (PwPs) via operationalized “virtual home tours.” By integrating quantitative usability data with qualitative insights, we aim to ultimately optimize this novel program as an effective, patient-centered vehicle for fall prevention in PD. This investigation strives to ensure that future interventions developed by the PI and tested in randomized, controlled studies are not only accessible and effective but also pragmatic and user-friendly.

## 2. Materials and Methods

This study is a sub-investigation of data from a single-site, single-arm, non-randomized pilot study evaluating a Principal Investigator (PI)-developed *novel virtual home safety intervention*. The sub-investigation focuses on feedback from participants—patients, CPs, and the study physical therapist—with respect to the virtual intervention. The parent study took place at the Movement Disorders Clinic of Rush University Medical Center, a large tertiary care center located in the Chicago metropolitan area, serving urban-, suburban-, and rurally located patients from Illinois and the surrounding Midwest states. The study was approved by the Rush University Institutional Review Board.

**Figure 1:**
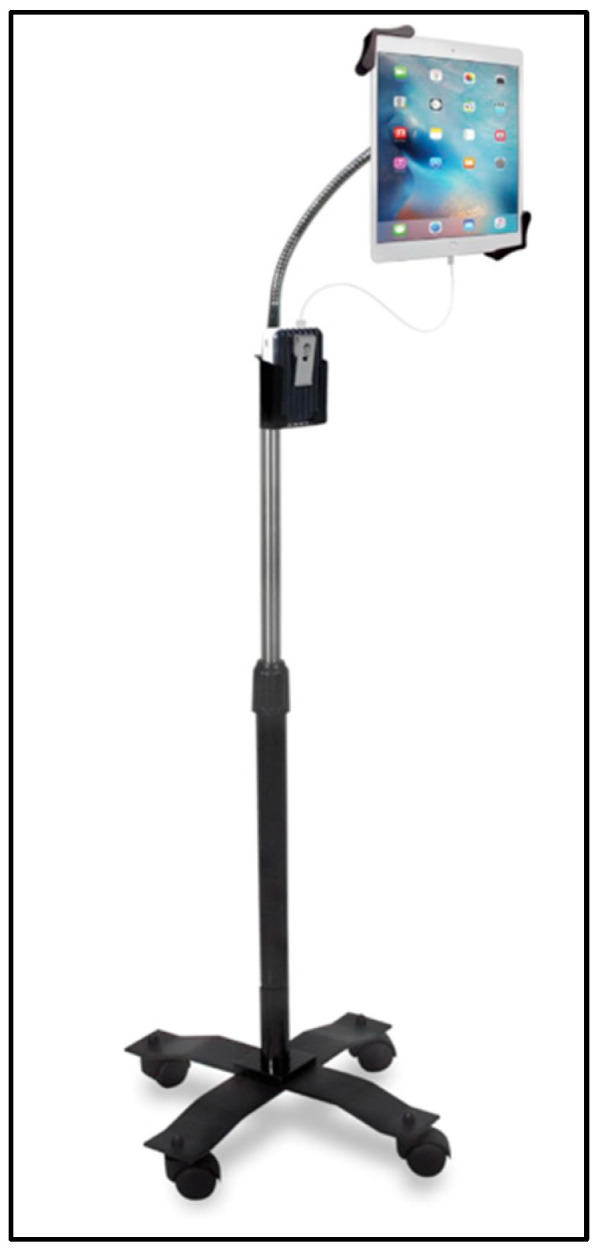
Mobile virtual platform.

The parent study involved a three-month intervention designed to reduce environmental fall risk factors for PwPs through guided and personalized home safety assessments (i.e., “virtual home tours”) delivered remotely by a physical therapist. The physical therapist was neurologically certified and was highly experienced in working with PwPs with a variety of gait and balance issues, including freezing-of-gait. Eligible PwPs had mild to moderate PD (Hoehn & Yahr Stage 2 and 3), were ambulatory without the need for assist devices but at risk for falls (had at least one near-fall or fall monthly in the preceding three months), and had a consenting CP who could be present at all televisits. Following an in-person baseline visit, each dyad (PwP + CP) participated in four video televisits with the therapist every four weeks (week 0, 4, 8, and 12), with an option to add two additional televisits at the discretion of the therapist (week 2, week 6). Dyads were provided a **mobile virtual platform (iPad tablet mounted on a rolling stand)** to facilitate the televisits and “virtual home tours” (Figure 1). This platform was hypothesized by the PI to allow for higher image stabilization and reduce dual-tasking on behalf of the patient who is inherently at risk for falls. Televisits on the tablet were accessed via a HIPAA-compliant Electronic Medical Record (EMR)-integrated video system (Vidyo Connect, Epic), the same platform by which patients would access televisits with their PD neurologists at Rush University. Televisits conducted by the therapist included virtual walk-throughs of the PwP’s home and identification of fall hazards using a standardized assessment tool provided to the dyad—a modified version of the validated Home Safety Self-Assessment Tool (mHSSAT) previously developed by Tomita et al. [5]. The original HSSAT has demonstrated benefits in reducing environmental fall risk factors in cohort studies, but has never been adapted to a virtual platform [5,6,7]. In this pilot study, the HSSAT was modified to be administered virtually and was used to operationalize “virtual home tours.” Like the original HSSAT, the mHSSAT offers specific solutions for fall risk factors identified from ten typical areas in a home (entrance to the front door yard, entrance to the back/side door, hallway or foyer, living room, kitchen, bedroom, bathroom, staircases, and laundry room/basement). The manual includes cartoon depictions of each area and potential fall risk factors, which are then numbered and correspond to potential solutions and other relevant chapters of the manual. These six chapters include: (1) general tips for in-home fall prevention and clutter reduction, (2) currently available useful products to prevent falls, (3) tables of regional home improvement service providers and vendors for durable medical equipment, (4) home modification guidelines (e.g., for grab bar installation), (5) common home improvement “how to’s,”, and (6) action logs for dyads to record and track progress towards recommendations made by the therapist. The therapist derives recommendations from and provides references to the manual as the dyad provides the “virtual home tour” during each televisit. The intervention did not include a systematic assessment of individual motor symptoms. The therapist evaluated environmental fall risk factors in the context of how patients functionally interacted with their space and the specific challenges they faced related to fall risk that included PD motor symptoms, namely bradykinesia, postural instability, and freezing-of-gait. This approach enabled tailored recommendations based on patient-specific and PD-related challenges.

Following the completion of the intervention at week 12, three surveys/questionnaires were administered electronically via REDCap to dyads to assess their overall satisfaction (see Supplement S1–S3), and one survey was administered to the study therapist (see Supplementary File S4). PwPs were encouraged to complete the surveys collaboratively with the CP.

### 2.1. Dyadic Survey 1: Satisfaction

A PI-developed survey, informed by existing telehealth satisfaction frameworks, consisting of 16 five-item Likert-based questions, was used to evaluate satisfaction across key domains, including the technical setup and connectivity, program quality, number of visits, privacy, safety, convenience, general usefulness, CP support, and overall experience (see Supplementary File S1) [8,9]. Likert responses ranged from *extremely satisfied* to *not satisfied at all* and from *strongly agree* to *strongly disagree*. Open feedback was also elicited to allow dyads to elaborate on their responses and provide additional qualitative insights.

### 2.2. Dyadic Survey 2: Preferences

A PI-developed survey consisting of four questions and ranked preference assessments was used to assess preferences with respect to (1) the technology used to perform virtual home safety evaluations (mobile virtual platform vs. tablet without a stand vs. smartphone), and (2) how they would prefer to receive home safety evaluations (virtual visits, in-home in-person visits, outpatient in-person visits) (see Supplementary File S2).

### 2.3. System Usability Scale (SUS): Dyadic Perceived Usability of the Mobile Platform

The System Usability Scale (SUS) is a widely used 10-item scale designed to quantify the usability of a system and is commonly used to assess digital tools [10]. The SUS is a balanced survey consisting of five questions with positive statements and five questions with negative statements, with scores ranging from 0 to 100. Questions on the SUS pertained to ease of use, user confidence, and perceived usability of the mobile virtual platform (see Supplementary File S3). A final SUS score of >80.3 indicates excellent usability, 69–80.3 good, 68 acceptable, 51–67 poor, and <51 awful.

### 2.4. Therapist Survey: Satisfaction and Limitations

In addition to participant feedback, the study therapist completed a PI-developed questionnaire pertaining to each dyad following their final televisit (week 12). This survey consisted of six Likert-based questions evaluating satisfaction across key domains, the technical setup, program quality, safety, convenience, and overall experience with the virtual home safety program for the particular dyad. These questions and Likert responses evaluating satisfaction mirrored the questions posed to the dyad in Dyadic Survey 1, with responses ranging from *extremely satisfied* to *not satisfied at all*. Open feedback was also elicited to allow the therapist to elaborate on the predominant limiting factors that prevented the dyad from fully benefiting from the program and implementing program recommendations. These factors were then tallied and thematically categorized. Open feedback responses were reviewed and thematically coded by two separate raters, the PI (MA) and the second author (SD). Codes were compared and discussed. Thematic saturation was considered achieved when no new themes emerged after reviewing the final set of responses.

### 2.5. Statistics

Descriptive statistics were used to summarize the responses. For Likert-based questions, responses were summarized by calculating the percentage of participants selecting each option. Ranking questions were summarized by frequency and percentage distribution. Open feedback responses from dyads and the therapist were manually reviewed and grouped into thematic categories based on content similarity, perceived benefits, challenges, and suggestions. The frequency of responses within each theme was then calculated to identify commonly cited issues and positive aspects of the intervention. Microsoft Excel and Chat GPT version 4 Omni were used for data analysis and visualization.

## 3. Results

### 3.1. Patient Demographics and Clinical Characteristics

Twenty-three dyads completed the study. The median age of PwPs was 68 [IQR 64–72.5] years, with 56.5% being male (Table 1). The cohort was predominantly White (95.7%), with 91.3% having at least some college education. All PwPs had mild to moderate disease severity (HY Stage 2–3) with a median MDS-UPDRS Part III score of 44 [IQR 27–52.5] and were at risk for falls (at least one fall or one near-fall monthly for preceding 3 months; no assistive devices). The median MoCA score was 27 [IQR 23.5–28], and disease duration was 7 [IQR 4.75–14] years. The majority of PwPs lived ≥25 miles from the clinic (65.3%).

### 3.2. Patient and Carepartner Satisfaction

**Figure 2:**
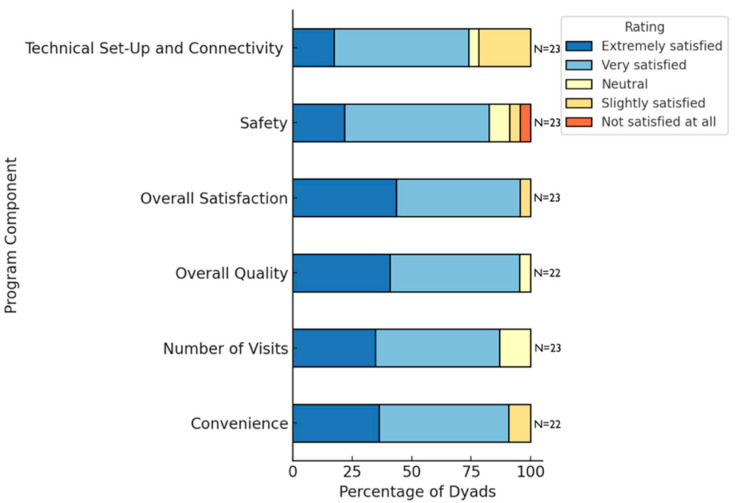
Patient and carepartner satisfaction with the virtual home safety program.

Figure 2 summarizes the results of questions from Dyadic Survey 1 regarding satisfaction, where we received responses from all 23 dyads, except for overall quality and convenience, where one response was missing for each. As evidenced in the figure, 95.65% (*n* = 22/23) of the dyads were *very* to *extremely satisfied* with the entire program overall (‘overall satisfaction’), with a median score of four (*very satisfied*) on the five-point Likert scale. In fact, for all of the other domains, the median score was four (*very satisfied*) on the five-point Likert scale. Of these domains, responses were most varied for technical setup and connectivity, and safety. One dyad was *not satisfied at all* with the safety of the program, quoting that *“the stand for the tablet was definitely a severe trip hazard.”* In Dyadic Survey 2, this same dyad chose in-home, in-person evaluations as their most preferred method of receiving home safety evaluations.

Almost all dyads, 95.65% (*n* = 22/23), agreed or strongly agreed with the statement “I learned a lot about unsafe practices in my home that could lead to falls that I had not realized before I had participated in the program.” All dyads (*n* = 23/23) agreed or strongly agreed with the statement “I would recommend this home program to all aging patients, whether they have Parkinson’s Disease or not.”

**Figure 3:**
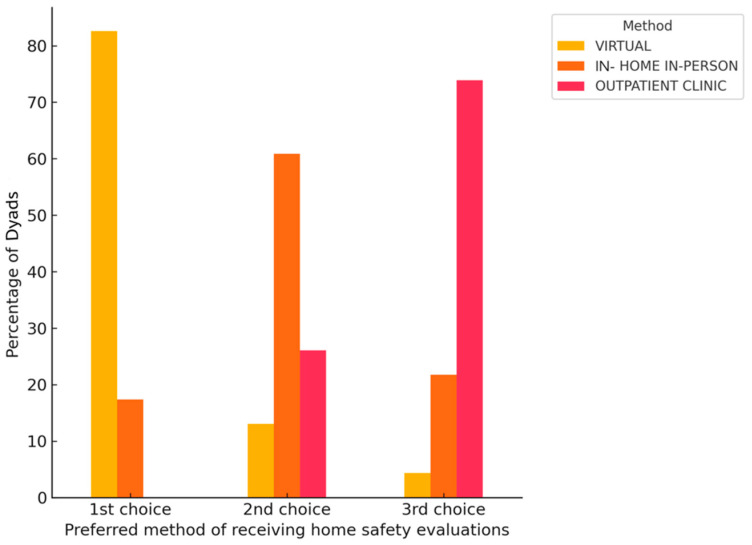
Dyadic preferences with respect to method of home safety evaluation (*n* = 23).

As visualized in Figure 3, when asked to rank their preferences for method of receiving home safety evaluations, 82.61% (*n* = 19/23) ranked a *virtual* home safety evaluation as their number one choice, followed by *in-home, in-person* evaluations (60.87%, *n* = 14/23). Zero dyads chose an *outpatient clinic*-based home safety program as their first choice.

**Figure 4:**
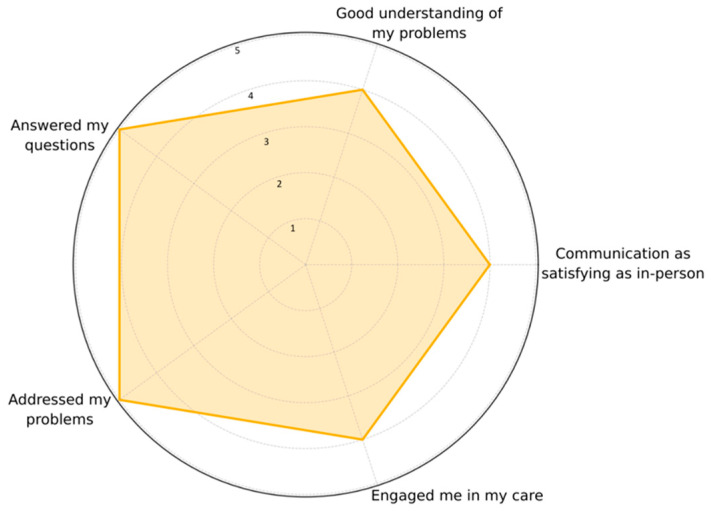
Dyadic satisfaction with virtual care provided by the study therapist (*n* = 23).

Dyads were asked if they agreed or disagreed with statements regarding communication with the study therapist and if they felt the therapist could gain a good understanding of their problems, answer their questions, address their problems, and engage them in their care virtually. Dyads were overwhelmingly happy with these elements of the virtual care provided by the study therapist, with a median score of four (*agree*) for all of these questions on the five-point Likert scale, as evident in Figure 4.

With respect to privacy, 82.61% (*n* = 19/23) of dyads *agreed* or *strongly agreed* that they were comfortable with the therapist “*coming into their home virtually.*” There were a few comments on open feedback that dyads had privacy concerns in that they disliked having strangers physically in their homes, and so they preferred a virtual program.

The role of the CP in the virtual home safety program was also further explored. Answers were varied with respect to the statement, *“I would have been able to participate in the home program on my own without a carepartner.”* In total, 47.82% (*n* = 11/23) *disagreed* or *strongly disagreed*, 13.04% (*n* = 3/23) were neutral, and 39.13% *agreed* or *strongly agreed* with the statement. However, 91.30% (*n* = 21/23) of patients *agreed* or *strongly agreed* with the statement, *“Having my carepartner participate in the visits with me enhanced the benefit of the program.”* Upon open feedback, patients felt that it was easier to do video visits together with their CPs—CPs helped them navigate the tablet and mobile platform, both with respect to technology and mobility. For example, one patient commented, *“Sometimes I did not have the mobility or flexibility in my hands to maneuver the iPad so my carepartner drove.”*

### 3.3. Patient and Carepartner Open Feedback

Table 2 summarizes the positive feedback from dyads, which have been thematically categorized with the frequency of responses indicated in parentheses. Dyads felt that the program helped them make positive changes towards fall prevention, and several stated that they would recommend it even for people without PD. Open feedback also revealed high satisfaction with the team, program content, convenience, and benefits to privacy afforded by the virtual home safety program. Using the mobile platform, connectivity issues, and using a tablet were the main challenges identified by dyads, in this order of frequency.

### 3.4. Therapist Satisfaction and Open Feedback

**Figure 5:**
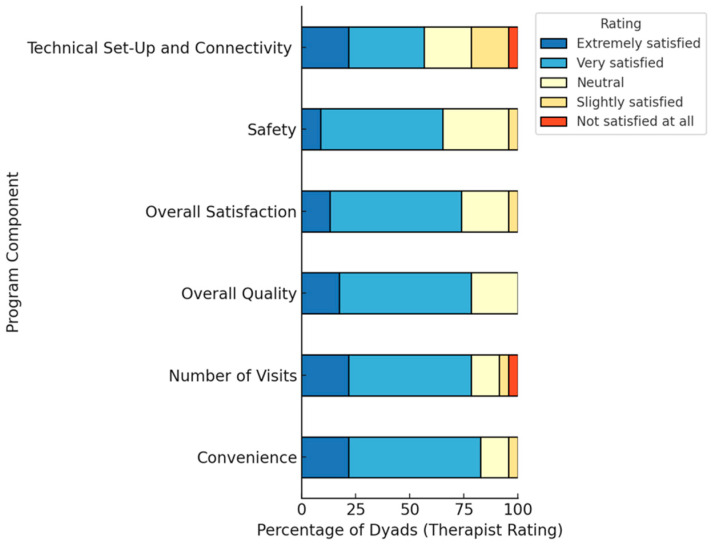
Therapist satisfaction with the virtual home safety program (*n* = 23).

Figure 5 summarizes the results of the Therapist Survey regarding satisfaction, where we received responses for all 23 dyads. As evidenced in the figure, the therapist reports she was *very* to *extremely satisfied* with the program for 73.91% (*n* = 17/23) of the dyads. In fact, for all of the domains, the median score was four (*very satisfied*) on the five-point Likert scale. Of these domains, responses were most varied for technical setup and connectivity, and the number of visits. For one dyad, there were frequent issues with audiovisual connectivity using broadband internet in their home at all of their televisits; however, the therapist was able to use Doximity Video Dialer to complete all of their televisits instead of VidyoConnect on Epic. Additionally, for one dyad, the therapist felt that, due to the patient’s cognitive difficulty, the dyad would have benefited from additional visits.

With respect to positive open feedback, notably, the study therapist identified the convenience of the televisits to be an important positive aspect of the program for this patient population. Given that the patient population had PD with consequent mobility issues and motor symptoms, the therapist felt that the virtual program was especially critical for at least two participants who were struggling with frequent motor “OFF”-levodopa symptoms of their PD and for several dyads who lived in remote rural areas.

**Figure 6:**
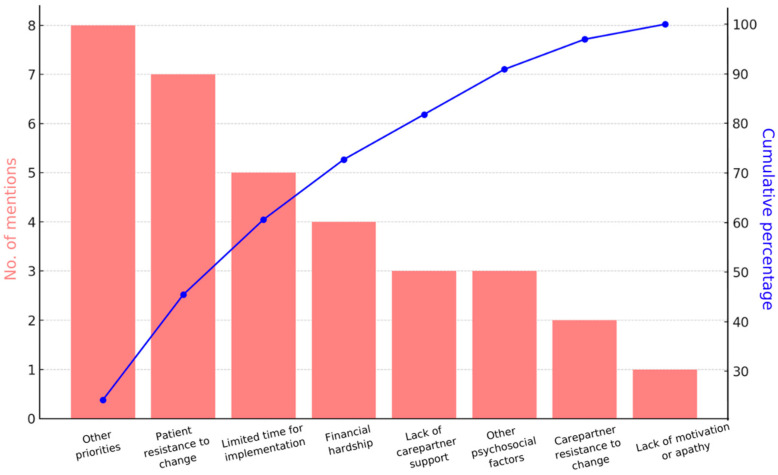
Limiting factors to optimal program benefit, as identified by the study therapist.

With respect to negative open feedback, Figure 6 is a Pareto chart that summarizes the frequency of mentions in descending order, represented by bars, and the cumulative percentage of the mentions represented by the curved line. As evident in the figure, ‘other priorities,’ followed by ‘patient resistance to change,’ and ‘limited time for implementation,’ accounted for 60% of the total number of limiting factors identified cumulatively for all 23 dyads. Other priorities included tending to mandatory home repairs (e.g., mold and water damage), urgent non-neurologic health issues, and urgent carepartner health issues. In terms of ‘patient resistance to change,’ patients struggled with seeing the benefits of making the recommended changes for fall prevention. For example, many patients struggled with breaking unsafe habits or preferences—one patient resisted decluttering his home workshop where he spent the majority of his waking hours, and another patient purchased furniture risers but felt they were “too ugly” to use. In terms of ‘limited time for implementation,’ many dyads had hired someone to perform repairs or install recommended items; however, this did not occur until after the end of the study. Of note, in terms of ‘other psychosocial factors,’ these included excessive pets, hoarding behaviors, and family conflicts.

### 3.5. Usability of the Technology and Dyadic Technological Preferences

**Figure 7:**
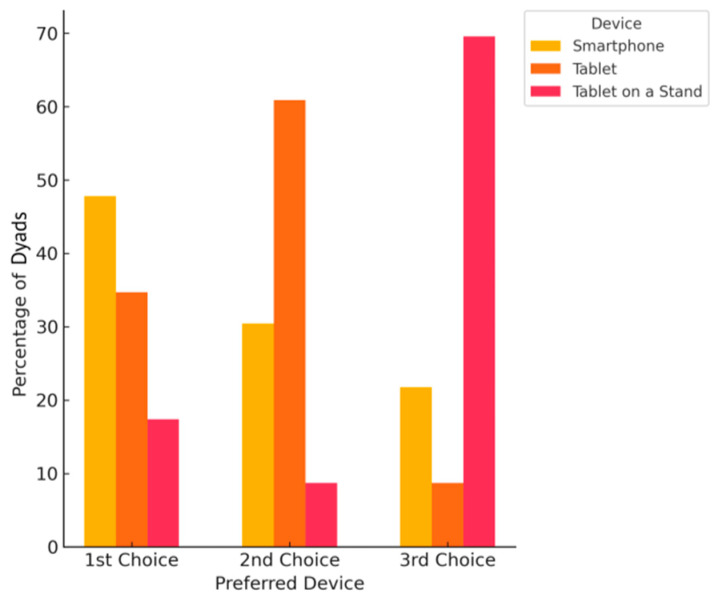
Dyadic preferences with respect to device for home safety evaluation (*n* = 23).

The mean SUS score for the mobile virtual platform (tablet mounted on a rolling stand) was 65 (*n* = 23/23), which corresponds to poor perceived usability. This finding was consistent with the aforementioned quantitative and qualitative feedback from the dyads, highlighting challenges with the mobile platform. On Dyadic Survey 2, we asked dyads if they agreed or disagreed with the statement, “*I think performing the virtual home safety evaluations with a smartphone would have been equivalent to the mobile platform*,” and 86.96% (*n* = 20/23) agreed. As visualized in Figure 7, we then asked dyads to rank their preferences for devices to participate in a virtual home safety program; 47.83% (*n* = 11/23) ranked a *smartphone* as their number one choice, followed by evaluations of a *tablet* alone (34.78%, *n* = 8/23). From this analysis, it was evident that the rolling stand itself proved to be problematic. Of note, all participants in the study owned a smartphone, and 60% owned their own tablet, so the majority of the participants had familiarity with both devices.

## 4. Discussion

In summary, our study revealed high overall satisfaction with the PI-developed *novel virtual home safety intervention* both from the perspective of the participating dyads and the study therapist—95.65% (*n* = 22/23) of the dyads were *very* to *extremely satisfied* with the entire program overall and the therapist indicated the same for 73.91% of the dyads (*n* = 17/23). While this paper focuses on stakeholder satisfaction and usability, data regarding the intervention’s impact on the primary clinical outcomes will be presented shortly in a forthcoming manuscript; however, preliminary analysis shows a median reduction in environmental fall hazards by 55.6% for the cohort. From the survey data alone, almost all dyads (95.65%, *n* = 22/23) *gained new awareness of home fall hazards from the program*. Dyads were satisfied with the virtual care they were provided by the study therapist, suggesting that this virtual format of identifying and addressing home safety concerns and engaging with the PwPs and their CPs is feasible and comparable to in-person care. Nearly all (91.30%) PwPs indicated that CP participation enhanced the benefit of the program. Given their central role, framing CPs as active participants rather than passive observers could enhance adherence and sustainability of the program recommendations. Future versions of this program may benefit from more integration of the CP with clearly defined expectations for their role in the virtual home tours and training modules of the mHSSAT manual. It could also be insightful to receive independent feedback from CPs and monitor their burden, as this could further enhance engagement and strengthen the overall effectiveness of the program.

Moreover, dyads expressed a clear preference for a telehealth-enabled home-based program to address home safety, as compared to an in-home, in-person, or outpatient clinic-based program. Through both quantitative and qualitative feedback, dyads felt that this was a program that would be beneficial for all aging patients, regardless of a PD diagnosis. In the qualitative feedback, more than one dyad commented on the evolving needs of aging patients with respect to home safety and how the virtual format of the program would be a convenient way to “revisit” those needs over time. Additionally, this analysis revealed a unique advantage of the virtual program: preserved privacy. More than one dyad was concerned about inviting strangers into their home, which would be necessary for a home-based care model.

Analysis of the survey data from the participating dyads and the study therapist was especially enlightening with respect to the digital tools used for this *novel virtual home safety intervention.* While 90/92 protocolized mandatory televisits were completed successfully by participants with zero adverse events, technology-related challenges consistently contributed to lower satisfaction ratings and negative open feedback shared from dyads and the therapist across the various surveys. The rolling tablet stand was chosen with the intention of image stabilization and simplifying “virtual home tours” in a study population that is inherently at risk for falls; however, it paradoxically proved more problematic and even a potential source of fall risk. From the open feedback, dyads experienced a diverse array of challenges with the rolling tablet stand, including difficulty navigating it, some indicating wheels did not move smoothly, and that it was “cumbersome.” Some dyads indicated a lack of familiarity with a tablet, in this case an iPad, and irregular audiovisual connectivity was a drawback to the program. Responses from dyads to various questions indicated that a smartphone could be equally, if not more, effective due to familiarity and its handheld nature. Putting this information together, for future home-based virtual programs, smartphones may be a better solution because of (1) the increasing ubiquity of these devices even amongst aging patients, and (2) the ability to rely on mobile data instead of broadband internet for the audiovisual connection. In fact, when we looked at the details of each televisit, we found that for seven dyads over the course of 10 televisits, the therapist resorted to Doximity Video Dialer to connect to the dyad on their smartphones due to connectivity issues. For these televisits, dyads were presumably using their mobile data, and televisits were completed successfully without connectivity issues. Additionally, for future studies, including a baseline virtual training session for participants to further familiarize themselves with the various digital tools and the telehealth platform, and to test the connection, would be beneficial. To improve usability and satisfaction, future programs should allow participants to choose from a range of device options based on their individual comfort and needs. Moreover, future studies could also be designed to compare connectivity quality in televisits within EMR-integrated video systems (Vidyo Connect, Epic), as used in this pilot study, versus third-party platforms like Doximity Video Dialer.

### 4.1. Methodological Study Limitations

The surveys were developed specifically for this novel intervention by the PI and were not formally validated or pilot tested. However, survey development was heavily guided by the prior literature on validated telehealth satisfaction and usability surveys. Items were reviewed for content relevance and clarity. While all therapist ratings and observations were provided by a single experienced neurologically certified physical therapist, this may limit external validity and generalizability of provider perspectives. Incorporating multiple providers in future studies would offer broader insights. The study would also benefit from having independent feedback from the CPs, as this could monitor their burden and could further enhance engagement and strengthen the overall effectiveness of the program.

### 4.2. Interpretive Study Limitations

The study sample was relatively homogeneous, consisting primarily of White, college-educated and tech-literate individuals, which limits the generalizability of our findings to more diverse or underserved and socioeconomically disadvantaged populations. Future studies should seek to enroll more racially and educationally diverse participants to evaluate the program’s usability and acceptability across broader demographics.

## Figures and Tables

**Table 1:** Baseline patient demographics and clinical characteristics.

Category	*n* (%)
Age	
<65 y	6 (26.1)
≥65 y	17 (73.9)
*Median [IQR]*	*68 [64–72.5]*
**Sex**	
Male	13 (56.53)
Female	10 (43.47)
**Race**	
White	22 (95.65)
Other	1 (4.35)
**Highest level of education**	
Secondary/High School	2 (8.69)
Some College/University	5 (21.73)
College/University	7 (30.44)
Graduate Education	9 (39.14)
**Distance from Clinic**	
1–10 miles	2 (8.7)
10–24 miles	6 (26.1)
≥25 miles	15 (65.2)
*Median [IQR]*	*37 [15.5–100.5]*
**Community Type**	
Urban	6
Suburban	16
Rural	1
**Out-of-state vs. In-state**	
Out-of-state	4 (3 IN, 1 WI)
In-state	19 (IL)
**HY Stage**	
Stage 2	12
Stage 3	11
**Disease Duration**	
0–4 y	6 (26.1)
≥5 y	17 (73.9)
*Median [IQR]*	*7 [4.75–14]*
**MDS-UPDRS Part III score**	
1–19	2
20–38	6
≥39	15
*Median [IQR]*	*44 [27–52.5]*
**MoCA score**	
0–25	8 (34.78)
≥26	15 (65.22)
*Median [IQR]*	*27 [23.5–28]*
**LEDD**	
0–100 mg/dL	1 (4.2)
101–399	2 (8.3)
400–999	13 (54.2)
≥1000	7 (33.3)
*Median [IQR]*	*712 [575–1037.5]*

**Table 2:** Patient and carepartner open feedback.

Positive Feedback	
Theme (Frequency of Responses)	Example Statements
Appreciated positive changes for fall prevention (9)	*“Great program! I had no idea about all the everyday fall risks.”* *“We made the appropriate fall prevention changes, and our home is much safer now.”* *“I think many patients would benefit from a home evaluation for fall prevention! As we age we are not always aware of home hazards. The book was very informative!”* *“We think everyone should have this opportunity as part of their care plan as things evolve and an opportunity to revisit when needs change. This could reduce the falls and potential concussions and injuries.”*
The program was convenient (6)	*“It would be easier to comply with virtual.”* *“You don’t have to leave your home to drive to the clinic. The home safety evaluation is taking place in my home.”*
Satisfied with the team (6)	*“Everyone who helped on this program was able to provide valuable insights and was very professional.”* *“Therapist was clear and precise in giving advice to me.”*
Satisfied with the program content (5)	*“The content of the program was excellent.”* *“I like for the person to see the house and give suggestions based on what they see.”* *“I think it is important for the therapist to get the visuals of the home situation.”* *“It was beneficial having a 3rd party point out what we need to do to be safer in the home. It was nice to have a team to work with.”* *“Good home recommendations to remedy possible accidents.”*
Privacy (2)	*“Virtual is best because we don’t like strangers in our home.”*
**Negative Feedback**	
**Theme (frequency of responses)**	**Example statements**
Difficulty using the stand (13)	*“The table stand was cumbersome.”* *“The wheels on the cart did not always move so smoothly and I had to do a lot of tilting.”* *“The stand did not allow as much flexibility or ease showing some areas of my home.”* *“The stand was just awful! I almost fell down the basement steps trying to get it down there to show my desk and office…My wife took the iPad and walked it down both areas to show the PT. My smartphone would have been so much easier.”*
Connectivity issues (7)	*“The audio would break in and out.”* *“The connection was always bumpy.”* *“The only problem we encountered was there were times when the audio would break in and out. Don’t really know if that was on our end and where we were in our house.”* *“There were some challenges early on with login and with our microphone, but both improved outright, or were resolved during the course of the study.”*
Difficulty using the tablet (6)	*“Tablet is too bulky.”* *“I can use our smartphone, but I wasn’t used to the hospital iPad.”* *“This was a first experience with a tablet for us.”*

## Data Availability

The data presented in this study are available on request from the corresponding author due to privacy. During the preparation of this manuscript, the authors used ChatGPT 4o for the purposes of generating graphics and text editing. The authors have reviewed and edited the output and take full responsibility for the content of this publication.

## Supplementary Materials

The following supporting information can be downloaded at: https://www.mdpi.com/article/10.3390/jcm14145031/s1, File S1: Dyadic Survey 1: Satisfaction; File S2: Dyadic Survey 2: Technological Preferences; File S3: Therapist Survey: Satisfaction and Limitations; File S4: System Usability Scale: Dyadic Perceived Usability of the Mobile Platform.

## Abbreviations

The following abbreviations are used in this manuscript:

PD	Parkinson’s Disease
PwP	Person with Parkinson’s Disease
CP	Carepartner
