# Supplementary material for: Participant and Provider Perspectives on a Novel Virtual Home Safety Program for Fall Prevention in Parkinson’s Disease
Source: J Clin Med. 2025 Jul 16;14(14):5031. doi: 10.3390/jcm14145031 (PMC12295064; doi:10.3390/jcm14145031)
Supplement: Supplementary file 1 [file jcm-14-05031-s001.zip › Supplement S1.pdf]

Dyadic Survey 1: Satisfaction

Please fill out this survey with your care partner who attended the televisits with you and provide answers you agree upon. Please tell us about your satisfaction and agreement/disagreement with the following statements regarding the virtual home safety evaluation program.

a) Technical Support

1. How satisfied were you with the technical setup and quality of the virtual connection?

- ☐ Not satisfied at all
- ☐ Slightly satisfied
- ☐ Neutral
- ☐ Very satisfied
- ☐ Extremely satisfied

b) Program Quality

1. How satisfied were you with the overall quality of the virtual home safety program you received?

- ☐ Not satisfied at all
- ☐ Slightly satisfied
- ☐ Neutral
- ☐ Very satisfied
- ☐ Extremely satisfied

2. How satisfied were you with the overall number of visits of the virtual home safety program you received?

- ☐ Not satisfied at all
- ☐ Slightly satisfied
- ☐ Neutral
- ☐ Very satisfied
- ☐ Extremely satisfied

3. The therapist could get a good understanding of my medical problems during the video visits.

- ☐ Strongly disagree
- ☐ Disagree
- ☐ Neither Agree or Disagree
- ☐ Agree
- ☐ Strongly Agree

4. The therapist answered my questions.

- ☐ Strongly disagree
- ☐ Disagree
- ☐ Neither Agree or Disagree
- ☐ Agree
- ☐ Strongly Agree

5. The therapist addressed my problems.

- ☐ Strongly disagree
- ☐ Disagree
- ☐ Neither Agree or Disagree
- ☐ Agree
- ☐ Strongly Agree

6. The therapist engaged me in my care.

- ☐ Strongly disagree
- ☐ Disagree
- ☐ Neither Agree or Disagree
- ☐ Agree
- ☐ Strongly Agree

7. Talking to the therapist during a video visit is as satisfying as talking in person

- ☐ Strongly disagree
- ☐ Disagree
- ☐ Neither Agree or Disagree
- ☐ Agree
- ☐ Strongly Agree

c) Privacy & Safety

1. I felt comfortable with the therapist "coming into my home" virtually.

- ☐ Strongly disagree
- ☐ Disagree
- ☐ Neither Agree or Disagree
- ☐ Agree
- ☐ Strongly Agree

2. How satisfied were you with the safety of using the mobile platform to perform the virtual home safety evaluations?

- ☐ Not satisfied at all
- ☐ Slightly satisfied
- ☐ Neutral
- ☐ Very satisfied
- ☐ Extremely satisfied

d) Convenience

1. How satisfied were you with the convenience of the virtual home safety program you received?

- ☐ Not satisfied at all
- ☐ Slightly satisfied
- ☐ Neutral
- ☐ Very satisfied
- ☐ Extremely satisfied

e) General Usefulness

1. I learned a lot about unsafe practices in my home that could lead to falls that I had not realized before I had participated in this program.

- ☐ Strongly disagree
- ☐ Disagree
- ☐ Neither Agree or Disagree
- ☐ Agree
- ☐ Strongly Agree

2. I would recommend this home program to all aging patients, whether they have Parkinson's Disease or not.

- ☐ **Strongly disagree**
- ☐ **Disagree**
- ☐ **Neither Agree or Disagree**
- ☐ **Agree**
- ☐ **Strongly Agree**

f) Carepartner Support

1. I would have been able to participate in the home program on my own without my carepartner.

- ☐ **Strongly disagree**
- ☐ **Disagree**
- ☐ **Neither Agree or Disagree**
- ☐ **Agree**
- ☐ **Strongly Agree**

2. Having my carepartner participate in the visits with me enhanced the benefit of the home program.

- ☐ **Strongly disagree**
- ☐ **Disagree**
- ☐ **Neither Agree or Disagree**
- ☐ **Agree**
- ☐ **Strongly Agree**

g) Overall Experience

1. How satisfied were you <u>overall</u> with the entire virtual home safety evaluation program?

- ☐ **Not satisfied at all**
- ☐ **Slightly satisfied**
- ☐ **Neutral**
- ☐ **Very satisfied**
- ☐ **Extremely satisfied**

Please provide both positive and negative feedback, we want to hear everything, and details are important. If you are 'not satisfied' or 'slightly satisfied' or you 'disagree' or 'strongly disagree' with any of the above, please make sure to elaborate.
