# Supplementary material for: Participant and Provider Perspectives on a Novel Virtual Home Safety Program for Fall Prevention in Parkinson’s Disease
Source: J Clin Med. 2025 Jul 16;14(14):5031. doi: 10.3390/jcm14145031 (PMC12295064; doi:10.3390/jcm14145031)
Supplement: Supplementary file 1 [file jcm-14-05031-s001.zip › Supplement S2.pdf]

Dyadic Survey 2: Technological Preferences

Please fill out this survey with your care partner who attended the televisits with you and provide answers you agree upon.

We are considering whether patients can just use only their smartphones or tablets without a mobile stand, instead of the tablet on the mobile stand to participate in this program. This could allow for greater access to the program. The following questions are relevant to this topic. Please tell us if you agree or disagree with the following statements.

1. I think performing the virtual home safety evaluations with a smartphone would have been equivalent to the mobile platform (tablet + tablet stand on wheels).

☐ **Agree**
☐ **Disagree**

2. I think performing the virtual home safety evaluations with a tablet alone would have been equivalent to the mobile platform (tablet + tablet stand on wheels).

☐ **Agree**
☐ **Disagree**

3. In order of preference, 1st being the most preferred and 3rd being the least preferred, please rank which device you would prefer to use to participate in the virtual home safety program.

	1st	2nd	3rd
Smartphone	☐	☐	☐
Tablet without a stand	☐	☐	☐
Tablet with a stand (Mobile Platform)	☐	☐	☐

Please briefly explain your reasoning behind the preference indicated above.

____

4. In order of preference, 1st being the most preferred and 3rd being the least preferred, please rank how you would prefer to receive home safety modification recommendations for your home.

	1st	2nd	3rd
Virtual visits	☐	☐	☐
In-home visits	☐	☐	☐
Outpatient visits	☐	☐	☐

Please briefly explain your reasoning behind the preference indicated above.

____

Please provide any final feedback or commentary, whether positive or negative, here. Any feedback in helpful in evaluating and improving this program. Details are helpful.

____
