# Supplementary material for: Participant and Provider Perspectives on a Novel Virtual Home Safety Program for Fall Prevention in Parkinson’s Disease
Source: J Clin Med. 2025 Jul 16;14(14):5031. doi: 10.3390/jcm14145031 (PMC12295064; doi:10.3390/jcm14145031)
Supplement: Supplementary file 1 [file jcm-14-05031-s001.zip › Supplement S3.pdf]

# Therapist Survey : Satisfaction and Limitations

**Please tell us about your satisfaction with the virtual home safety evaluation program for this patient and carepartner pair.**

**1. During the televisits with this pair, how satisfied were you with the technical quality of the virtual connection?**

- ☐ Not satisfied at all
- ☐ Slightly satisfied
- ☐ Neutral
- ☐ Very satisfied
- ☐ Extremely satisfied

**2. During the televisits with this pair, how satisfied were you with the overall quality of the virtual home safety program you provided?**

- ☐ Not satisfied at all
- ☐ Slightly satisfied
- ☐ Neutral
- ☐ Very satisfied
- ☐ Extremely satisfied

**3. For this pair, how satisfied were you with the overall number of televisits you provided the virtual home safety program you provided?**

- ☐ Not satisfied at all
- ☐ Slightly satisfied
- ☐ Neutral
- ☐ Very satisfied
- ☐ Extremely satisfied

**4. For this pair, how satisfied were you with the safety of using the mobile platform to perform the virtual home safety evaluations?**

- ☐ Not satisfied at all
- ☐ Slightly satisfied
- ☐ Neutral
- ☐ Very satisfied
- ☐ Extremely satisfied

**5. For this pair, how satisfied were you with the convenience of the virtual home safety program you provided?**

- ☐ Not satisfied at all
- ☐ Slightly satisfied
- ☐ Neutral
- ☐ Very satisfied
- ☐ Extremely satisfied

**6. For this pair, how satisfied were you overall with the entire virtual home safety program?**

- ☐ Not satisfied at all
- ☐ Slightly satisfied
- ☐ Neutral
- ☐ Very satisfied
- ☐ Extremely satisfied

**Please provide both positive and negative feedback, we want to hear everything, and details are important. If you were 'not satisfied' or 'slightly satisfied' with any of the above, please make sure to elaborate on why you felt that was the case for this pair.**

______________________________________________________________________________

**7. For this pair, what was the predominant limiting factors as to why they did not abide by the home safety recommendations you made?**

**Here are some examples of potential limiting factors:**

**1. Patient resistance**
**2. Complex risk factors like pets, hoarding**
**3. Financial restraints/Lack of resources to make changes**
**4. Other priorities (health issues, construction of house, insurance issues, social obligations)**
**5. Caregiver resistance/unavailability**
**6. Limited time for implementation**

***Subject id*** ***1st Limiting factor*** ***2nd Limiting factor*** ***Please elaborate***

______________________________________________________________________________

**8. For this pair, who do you believe had the ultimate decision-making power as to making changes in the home?**
**1. Patient**
**2. Carepartner**
**3. Patient & Carepartner in fairly equal proportion**
**Please elaborate.**

***Therapist Overall Open Feedback:***

**Please provide any additional feedback or commentary, whether positive or negative, here regarding the following elements of the program for this specific patient and carepartner pair and overall. Any feedback is helpful in evaluating and improving this program. Details are helpful.**
