# Supplementary material for: Participant and Provider Perspectives on a Novel Virtual Home Safety Program for Fall Prevention in Parkinson’s Disease
Source: J Clin Med. 2025 Jul 16;14(14):5031. doi: 10.3390/jcm14145031 (PMC12295064; doi:10.3390/jcm14145031)
Supplement: Supplementary file 1 [file jcm-14-05031-s001.zip › Supplement S4.pdf]

System Usability Scale (SUS): Dyadic Perceived Usability of the Mobile Platform

In this survey, you will be responding to whether you agree or disagree to the following statements about the mobile platform used in the research study. The mobile platform is the tablet plus the tablet stand on wheels that was used during your televisits. Please complete the survey below.

1. I think that I would like to use the mobile platform frequently.

- ☐ Strongly disagree
- ☐ Disagree
- ☐ Neither Agree or Disagree
- ☐ Agree
- ☐ Strongly Agree

2. I found the mobile platform unnecessarily complex.

- ☐ Strongly disagree
- ☐ Disagree
- ☐ Neither Agree or Disagree
- ☐ Agree
- ☐ Strongly Agree

3. I thought the mobile platform was easy to use.

- ☐ Strongly disagree
- ☐ Disagree
- ☐ Neither Agree or Disagree
- ☐ Agree
- ☐ Strongly Agree

4. I think that I would need the support of a professional to be able to use the mobile platform.

- ☐ Strongly disagree
- ☐ Disagree
- ☐ Neither Agree or Disagree
- ☐ Agree
- ☐ Strongly Agree

5. I found the various functions of the mobile platform well integrated.

- ☐ Strongly disagree
- ☐ Disagree
- ☐ Neither Agree or Disagree
- ☐ Agree
- ☐ Strongly Agree

6. I thought there was too much inconsistency in this mobile platform.

- ☐ Strongly disagree
- ☐ Disagree
- ☐ Neither Agree or Disagree
- ☐ Agree
- ☐ Strongly Agree

7. I would imagine that most people would learn to use this mobile platform very quickly.

- ☐ **Strongly disagree**
- ☐ **Disagree**
- ☐ **Neither Agree or Disagree**
- ☐ **Agree**
- ☐ **Strongly Agree**

8. I found the mobile platform very awkward to use.

- ☐ **Strongly disagree**
- ☐ **Disagree**
- ☐ **Neither Agree or Disagree**
- ☐ **Agree**
- ☐ **Strongly Agree**

9. I felt very confident using the mobile platform.

- ☐ **Strongly disagree**
- ☐ **Disagree**
- ☐ **Neither Agree or Disagree**
- ☐ **Agree**
- ☐ **Strongly Agree**

10. I needed to learn a lot of things before I could get going with this mobile platform.

- ☐ **Strongly disagree**
- ☐ **Disagree**
- ☐ **Neither Agree or Disagree**
- ☐ **Agree**
- ☐ **Strongly Agree**
